# Synthesis of Lipoamino Acids and Their Activity against Cerebral Ischemic Injury

**DOI:** 10.3390/molecules14104051

**Published:** 2009-10-12

**Authors:** Li-Yun Yao, Qi Lin, Yin-Yao Niu, Ke-Min Deng, Jian-Hua Zhang, Yang Lu

**Affiliations:** Institute of Pharmaceutical Research, Shanghai Jiao Tong University School of Medicine, Shanghai 200025, China

**Keywords:** lipoamino acids, neuroprotection, brain ischemia, oxygen-glucose deprivation

## Abstract

A series of lipoamino acids were synthesized and their neuroprotective effect against brain ischemia induced by oxygen-glucose deprivation (OGD) on rat cerebral slices was evaluated. Among these compounds, *N*-stearoyl-l-tyrosine (**4**), *N*-stearoyl-l-serine (**5**) and *N*-stearoyl-L-threonine (**6**) exhibited good neuroprotective activity. We found that the neuroprotective activity of lipoamino acids depended on the acyl group, the presence of a free carboxylic function and a free hydroxyl group at the branched chain of the amino acids. The results also showed that **5** was the most active compound, protecting rat brain slices against OGD as well as hydrogen peroxide (H_2_O_2_) insult at the range of 1–10 M.

## 1. Introduction

Cerebral ischemia would lead to brain damage in pathogenetic mechanisms including excitotoxicity, over production of free radicals, inflammation and apoptosis [1,2,3]. One of the challenging brain damage therapies is to inhibit the toxic pathways by activating endogenous neuroprotective system to stimulate cellular responses against the damage induced by severe ischemic events [4,5,6]. A variety of chemical agents, such as glumatate receptor antagonists, antioxidants, anti-inflammatory and anti-apoptotic agents, have been used to initiate neuroprotection by mediating the processes [7,8,9]. Despite aggressive investigations aimed at development of innovative neuroprotective drugs, few are in fact clinically effective [10].

**Figure 1:**
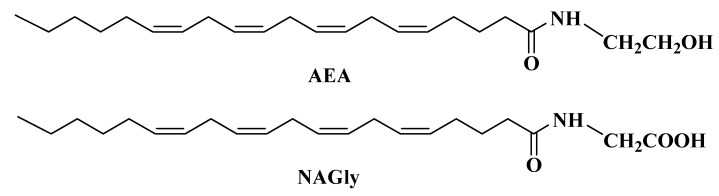
The structures of **AEA** and its endogenous analog (**NAGly)**.

Recent evidences suggest that *N*-arachidonoylethanolamine (anandamide, **AEA**, Figure 1), an endocannabinoid, plays a crucial neuroprotective role in certain neurodegenerative diseases [11,12,13]. Therefore, the design, synthesis and study of analogs of **AEA** with the aim of obtaining compounds with neuroprotective activity are ongoing research in our group. 

**Figure 2:**
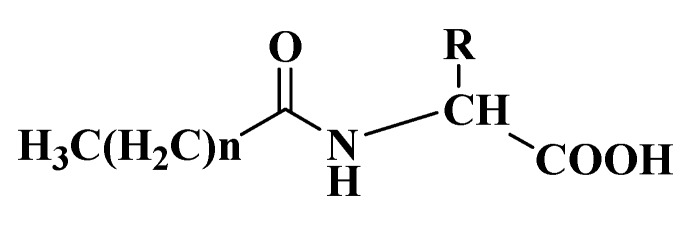
The general structure of **LA**s.

As chemical entities, lipoamino acids (**LA**s, Figure 2), the fatty acid-amino acid conjugates, exist as endogenous substances with multiple biological activities [14]. They appear to have close relationships with **AEA,** not only structurally, but also in terms of biological activities such as analgesia, anti-inflammatory effects, inhibition of cell proliferation, calcium ion mobilization and neural protection [15]. For example, *N*-arachidonoylglycine (**NAGly**, Figure 1) is an endogenous constituent in many tissues and might have analgesic properties similar to those reported for **AEA** [16]. Recently, we found that stearic acid protected rat brain slices or neurons *in vitro* against apoptosis induced by OGD, NMDA or H_2_O_2_ [17]. In terms of the structure and activity of both **LA**s and stearic acid, we deduced that **LA**s as amino acid conjugates with stearic acid might show neuroprotective activity. It is interesting to synthesize a series of *N*-stearoylamino acids and its analogues and evaluate their potential neuroprotective activity. By changing the structure and stereochemistry of the aminodonors, we would be able to find novel neuroprotective agents and fine-tune their SAR toward obtaining potent protectants. The present paper reports the synthesis of this library of **LA**s (Figure 2) and evaluation of their activity against cerebral ischemic injury *in vitro*. 

## 2. Results and Discussion

### 2.1. Chemical synthesis

**Scheme 1:**
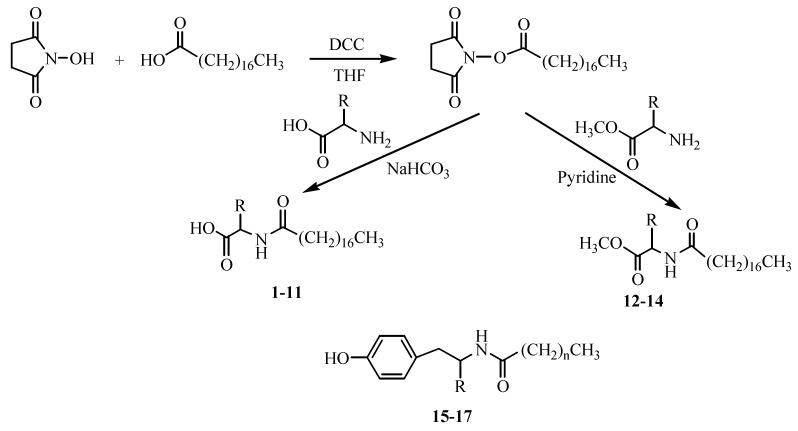
The synthetic rout to *N*-stearoylamino acids and their methyl esters.

A series of *N*-stearoylamino acids and their derivatives (Table 1) were synthesized using the procedures below, shown in Scheme 1 [18]. Briefly, commercially available stearic acid was reacted with *N*-hydroxysuccinimide to provide octadecanoic acid 2,5-dioxo-pyrrolidin-1-yl ester, which was combined with appropriate amino acids (or amino acid methyl esters) to give *N*-stearoylamino acids **1-11** and *N*-stearoylamino acid methyl esters **12-14**. The acylation of amine of amino acids with higher yield is especially suitable for the synthesis of **LA**s for its effective chemoselectivity towards the amino rather than hydroxyl group in the side-chains of amino acids. Compounds **15** and **16** were synthesized following the synthetic route of **4** from palmitic and lauric acids instead of stearic acid, respectively. Compound **17** was obtained by reducing **4** with NaBH_4_-I_2_ by the method reported by Mckennon *et al.* [19]. The NaBH_4_-I_2_ reagent system, by generating the reactive (acyloxy)borohydride (RCOOBH_2_), is more selective towards the carboxylic group rather than the amide group, because of the presence of the free carboxylic group in **LA**s [20]. The structures of all synthesized compounds were confirmed by ^1^H- and ^13^C-NMR, and the spectroscopic properties and analytical data were in accord with the proposed structures. 

### 2.2. Biological activity

#### 2.2.1. Effect of the synthesized compounds on OGD-insulted rat brain slices 

**Figure 3:**
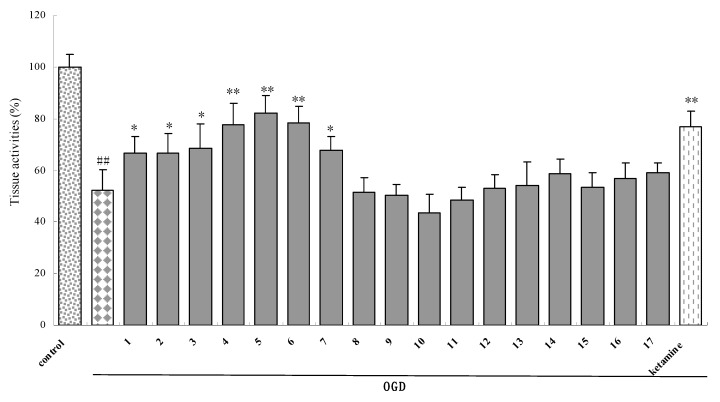
The tissue activity of rat brain slices pretreated with compounds **1-17** (10 μM) in the OGD-induced brain injury model. Brain slices were incubated with different compounds 30 min prior to and during OGD applications. The viability of brain slices was evaluated by using TTC staining method. Data are means ± SD from six independent experiments. ^##^ *P <0.01* vs control group. ^*^P <0.05, ^**^ *P <0.01* vs OGD group.

The compounds shown in Table 1 were screened for neuroprotective activity against brain ischemia induced by OGD insult on rat cerebral slices [21]. The screening concentrations of compounds were determined by our preliminary dose-response study (data not shown). The tissue activity of the brain slices was expressed as percentage of that of the control before brain injury. In Figure 3, the tissue activities of brain slices pretreated with **1-7** (66.5%–82.2%) were obviously higher than that of the brain slices pretreated with no drug (51.8%) (*P* < 0.05), indicating these compounds could reduce the OGD-induced neurotoxicity. Among these compounds, **4**, **5** and **6,** the three **LA**s with free hydroxyl groups, showed good tissue activity (77.5%, 82.2% and 76.5% respectively), which was similar to that of the standard ketamine (76.7%) and they attenuated OGD injury effectively (*P* < 0.01). It’s worthy to note that the pretreatment of the brain slices with target compounds did not influence the availability of brain ischemia model within the effective concentration (data not shown). Compound **4** showed a significant protective effect on insulted brain slices, but its homologues **15** and **16** had little effect (53.4%, 56.6% respectively), indicating that the length of the alkyl moiety of the **LA**s plays a critical role in the neuroprotective effect. *N*-stearoylamino acid methyl esters **12-14** and *N*-stearoyl-L-tyrosinol (**17**) presented little neuroprotective effect, implying that the presence of a free carboxylic group in the stearic amides may be necessary for their activity. It is interesting that an identical neuroprotective effect has been observed for **1** (L-phenylalanine) and **2** (DL-phenylalanine). A detailed SAR study concerning the stereochemistry of **LA**s and their neuroprotecive activity is currently under way in our laboratory. 

Brain slices not only maintain anatomic relations and natural synaptic connectivity *in vitro*, but also eliminate such *in vivo* variables as blood flow, temperature, ionic environment, and closely match *in vivo* conditions [22]. Therefore, increasing numbers of investigation used brain slice models have been reported to study brain function and brain protection. On the other hand, TTC method using 2,3,5-triphenyltetrazolium chloride (TTC) as an histopathologic stain for identification of infarcted rat brain tissue is simple, objective and sensitive, and has been widely used in the assessment of brain ischemia *in vitro* [23,24]. In the present study, we evaluated for the first time the effects of **LA**s on brain slice injury, using the TTC method.

#### 2.2.2. Effect of **5** on three types of insulted brain slices 

**Figure 4:**
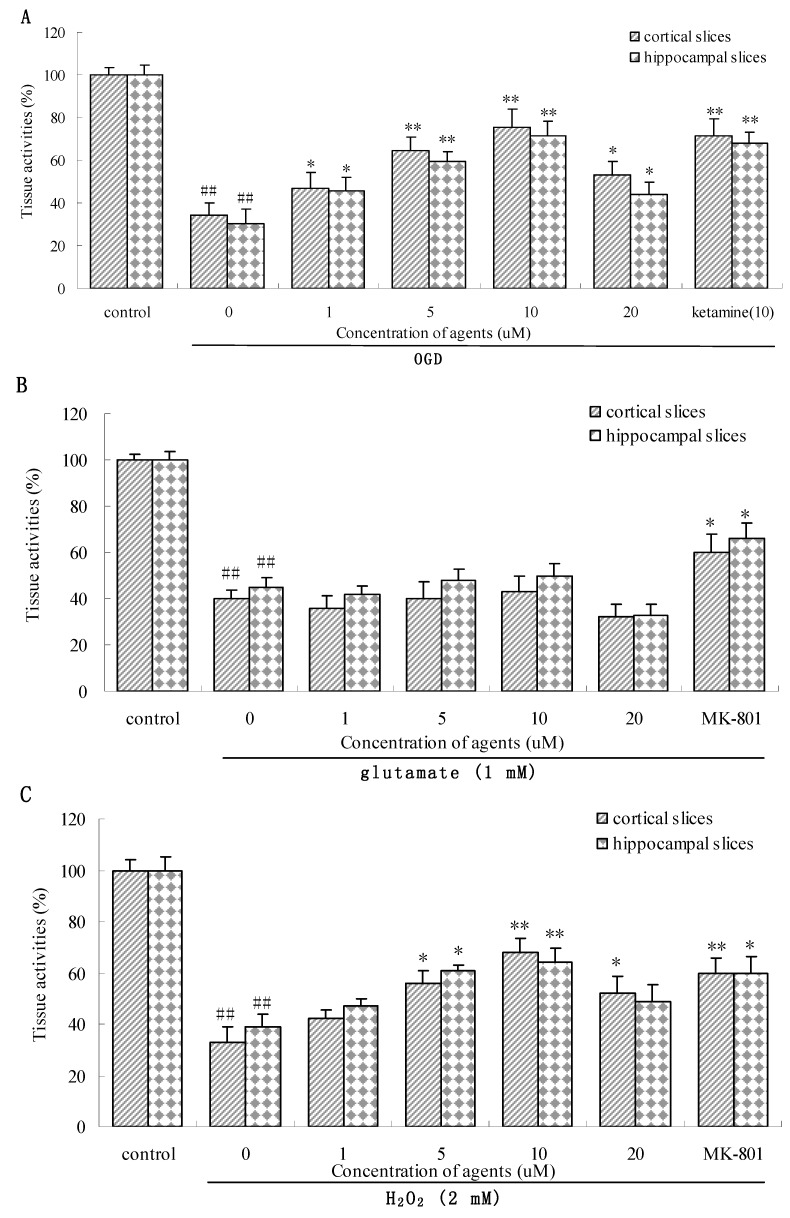
The tissue activity of rat brain slices pretreated with *N*-stearoyl-l-serine (**5**) on rat brain slices induced by OGD, Glu, or H_2_O_2_ insult. Brain slices were incubated with **5** 30 min prior to and during OGD (**A**), 1 mM glutamate (**B**), or 2 mM H_2_O_2_ (**C**) applications. The viability of cortical and hippocampal slices was evaluated by using TTC staining method. (n = 6). Data are mean ± SD. ^##^*P < 0.01* vs control group. ^*^P < 0.05, ^**^ *P < 0.01* vs injury group.

OGD, glutamate (Glu) or H_2_O_2_ damaged models can reflect the pathological characteristics of different phases of ischemia injury (metabolism disorder, toxic amino acid and oxidative stress) [25]. In order to evaluate the possible mechanism of neuroprotective action of these new molecules, we observed the neuroprotective effect of *N*-stearoyl-l-serine (**5**), the most effective one, against three types of damage brain cortical and hippocampal slices. As shown in Figure 4, compound **5** can concentration-dependently protect brain slices against OGD as well as H_2_O_2_ insult at the range of 1~10 μM (Figure 4A,C), and has no protective effect on 1 mM Glu injured brain slices (Figure 4B). The protective effect became less at 20 μM, implying that **5** at concentration higher than 20 μM might show the cytotoxicity.

Since the earlier consequences of applying OGD model in brain ischemia are both increasing release of the excitatory amino acid and excessive activation of oxidative free radicals [26], the observation that **5** might defend against oxidative stress and couldn’t inhibit the excitotoxicity of glutamate suggested that neuroprotective effect of **5** might be mediated, at least in part, by an anti-oxidative stress mechanism. These results are in agreement with our recent report showing that neuroprotective effect of *N*-stearoyl-l-tyrosine (**4**) may be attributed to reduction in lipid peroxidation and DNA fragmentation [27]. The mechanism studies of **4** and **5** could provide experimental foundation for investigating of their analogues. The detailed mechanism study of these compounds is currently under way in our laboratory.

## 3. Experimental 

### 3.1. General

Chemicals from Sinopharm Chemical Reagent Co. were used without further purification. Melting points were determined on a WRS-1A melting point apparatus and are uncorrected. Optical rotations were measured at 20±2°C with a Perkin-Elmer 241MC polarimeter. The progress of reactions was monitored by silica gel GF_254_ TLC plates. Column chromatography was performed on silica-gel 60 (100-200 mesh ASTM). ^1^H- and ^13^C-NMR spectra were recorded at 400 and 100 MHz, respectively, on a Bruker AM-400 spectrometer in chloroform-*d* solutions (unless stated otherwise), with TMS as internal standard. Chemical shifts were reported in ppm (δ) downfield from TMS. All the coupling constants (*J*) are in hertz. Mass spectra were recorded on an Agilent 1100 LC/MSD mass spectrometer. All reported yields correspond to yields of purified products.

### 3.2. Preparation of N-stearoylamino acids and their derivatives

#### 3.2.1. Synthesis and spectroscopic data of *N*-stearoylamino acids **1-11**

Dicyclohexylcarbodiimide (DCC, 9 g, 43.6 mmol) was added to a solution of stearic acid (10 g, 35.2 mmol) and *N*-hydroxysuccinimide (6 g, 52.1 mmol) in THF (80 mL) and the mixture was cooled in an ice-bath under stirring for 16 h [28]. After filtration, evaporation and recrystallization in isopropanol, octadecanoic acid 2,5-dioxo-pyrrolidin-1-yl ester was obtained with a yield of 90%, m.p. 76~78 °C. To a solution of the appropriate amino acid (5.5 mmol) and NaHCO_3_ (15 mmol) in water (20 mL) was added dropwise octadecanoic acid 2,5-dioxo-pyrrolidin-1-yl ester (5 mmol) in THF (20 mL). The mixture was stirring for 6 h at room temperature. The solvent was removed under reduced pressure. The residue was dissolved in water and adjusted with HCl (1 M) to pH 3, and the solution was extracted with ethyl acetate (2 times × 50 mL). The combined organic extracts were successively washed with water and saturated NaCl. Then the organic phase was dried over anhydr. Na_2_SO_4_ and evaporated to dryness. Compounds **1-6** and **8** were obtained through recrystallization from *n*-hexane, Compounds **10** and **11** from chloroform and **7** and **9** were purified by column chromatography over silica gel (eleuent: 40:1 EtOAc-CH_3_OH). 

*N-Stearoyl-L-phenylalanine* (**1**). Yield 80%; white solid; m.p. 66-68 °C; [α]${}_{D}^{21}$: +20.9° (*c* 0.92, CHCl_3_); ^1^H-NMR: δ (ppm) 7.27 (m, 3H of Ph), 7.15 (d, *J =* 6.6 Hz, 2H of Ph), 6.07 (d, 1H, *J =* 7.3 Hz, NH), 4.90 (t, 1H, *J* = 6.0 Hz, CHN), 3.13-3.23 (m, 2H, CH_2_Ph), 2.17 (t, 2H, *J =* 4 Hz, CH_2_CON), 1.55 (m, 2H, CH_2_), 1.25 (m, 28H, 14 × CH_2_), 0.88 (t, 3H, *J* =6.9 Hz, CH_3_); ^13^C-NMR: δ (ppm) 174.6, 174.1 (2C, acid and amide C=O), 135.7 (*i*-C of Ph), 129.3 (2 × *o*-CH of Ph), 128.6 (2 × *m*-CH of Ph), 127.2 (*p*-CH of Ph), 53.1 (CHN), 37.3 (CH_2_Ph), 36.4 (CH_2_-amide), 33.3(CH_2_), 31.9 (CH_2_), 29.7-29.2 (12 × CH_2_), 25.5 (CH_2_), 22.6 (CH_2_), 14.1 (CH_3_); ESI-MS: [M-1]^+^ m/z 430.1.

*N-Stearoyl-DL-phenylalanine* (**2**). Yield 67%; white solid; m.p. 93-95 °C; [α]${}_{D}^{21}$: 0° (*c* 0.92, CHCl_3_); ^1^H-NMR and ^13^C-NMR spectra were identical to those of **1**; ESI-MS: [M-1]^+^ m/z 430.1.

*N-Stearoyl-L-proline* (**3**). Yield 50%; white solid; m.p. 87-89 °C; [α]${}_{D}^{22}$: +32.1° (*c* 1.0, CHCl_3_); ^1^H-NMR: δ (ppm) 4.81-4.66 (m, 5H of pyrrolidine), 3.22 (m, 2H of pyrrolidine), 2.13 (t, 2H, *J* = 3.5 Hz, CH_2_CON), 1.55 (m, 2H, CH_2_), 1.23 (m, 28H, 14 × CH_2_), 0.89 (t, 3H, *J =* 7 Hz, CH_3_); ^13^C-NMR: δ (ppm) 174.7, 174.2 (2 × C=O, acid and amide), 61.7 (N-CH of pyrrolidine), 53.4 (N-CH_2_ of pyrrolidine), 37.3(CH_2_), 36.4 (CH_2_-amide), 31.9 (CH_2_), 29.7-29.1(13 × CH_2_), 25.5 (CH_2_-pyrrolidine), 22.6 (CH_2_-pyrrolidine), 14.1 (CH_3_); ESI-MS: [M+Na]^+^ m/z 404.2. 

*N-Stearoyl-L-tyrosine* (**4**). Yield 50%; white solid; m.p. 134-136 °C; [α]${}_{D}^{18}$: +41.7° (*c* 1.0, CH_3_OH); ^1^H-NMR: δ (ppm) 6.99 (d, *J =* 10.4 Hz, 2H of Ph), 6.73 (d, *J =* 10.2 Hz, 2H of Ph), 5.94 (s, 1H, NH), 4.82 (t, 1H, *J =* 6.0 Hz, CHN), 3.1 (m, 2H, CH_2_Ph), 2.17 (t, 2H, *J =* 7.8 Hz, CH_2_CON), 1.56 (m, 2H, CH_2_), 1.26 (m, 28H, 14 × CH_2_), 0.88 (t, 3H, *J =* 6.8 Hz, CH_3_); ^13^C-NMR: δ (ppm) 174.1,173.9 (2 × C=O, acid and amide), 155.3 (C-OH of Ph), 133.5(*i*-C of Ph),130.4 (2 × *o*-CH of Ph), 115.8 (2 × *m*-CH of Ph), 53.4 (CHN), 36.8 (CH_2_Ph), 36.5 (CH_2_-amide), 31.9 (CH_2_), 31.5 (CH_2_), 29.7-29.2 (12 × CH_2_), 25.6 (CH_2_), 22.6 (CH_2_), 14.0 (CH_3_); ESI-MS: [M+1]^+^ m/z 448.2.

*N-stearoyl-L-serine* (**5**). Yield 60%; white solid; m.p. 100-102 °C; [α]${}_{D}^{19}$: +21.2° (*c* 1.0, CH_3_OH); ^1^H-NMR (CD_3_OD): δ (ppm) 4.48 (t, 1H, *J =* 4.4 Hz, N-CH), 3.86 (m, 2H, CH_2_O), 2.26 (t, 2H, *J =* 7.4 Hz, CH_2_CON), 1.62 (m, 2H, CH_2_), 1.28 (m, 28H, 14 × CH_2_), 0.89 (t, 3H, *J =* 6.8 Hz, CH_3_); ^13^C-NMR (CD_3_OD): δ (ppm) 176.7, 173.8 (2 × C=O, acid and amide), 63.3 (CH_2_O), 56.3 (CHN), 37.2 (CH_2_-amide), 33.3 (CH_2_), 31.1-30.6 (12 × CH_2_), 27.1 (CH_2_), 24.0 (CH_2_), 14.7 (CH_3_); ESI-MS: [M+1]^+^ m/z 372.1.

*N-Stearoyl-L-threonine* (**6**). Yield 56%; white solid; m.p. 62-64 °C; [α]${}_{D}^{22}$: +12.5° (*c* 0.92, CH_3_OH); ^1^H-NMR (CD_3_OD): δ (ppm) 6.8 (s, 1H, NH), 4.51 (d, 1H, *J* = 7.8 Hz, CHN), 4.43 (s, 1H, CHO), 2.3 (t, 2H, *J* = 7.4 Hz, CH_2_CON), 1.63 (d, 2H, *J* = 6.7 Hz, CH_2_), 1.25 (m, 28H, 14 × CH_2_), 1.21 (d, 3H, *J* = 6.0 Hz, CH_3_), 0.88 (dd, 3H, *J_1_* = 6.7, *J_2_* = 7.0 Hz, CH_3_); ^13^C-NMR (CD_3_OD): δ (ppm) 177.2, 174.1 (2 × C=O , acid and amide), 66.5 (CHO), 59.6 (CHN), 36.9 (CH_2_-amide), 34.2 (CH_2_), 30.3-29.6 (12 × CH_2_), 26.1 (CH_2_), 23.3 (CH_2_), 17.5 (CH_3_), 14.1 (CH_3_); ESI-MS: [M+1]^+^ m/z 389.2.

*N-Stearoyl-L-tryptophan* (**7**). Yield 30%; brown solid; m.p. 88-90 °C; [α]${}_{D}^{20}$: +28° (*c* 1.0, CHCl_3_); ^1^HNMR: δ (ppm) 8.37 (s, 1H, NH of indole), 7.54 (d, 1H, *J* = 7.8 Hz, N-CH of indole), 7.3-6.96 (m, 4H, Ph-H of indole), 6.14 (d, 1H, *J* = 7.6 Hz, NH), 4.93 (m, 1H, CHN), 3.32 (m, 2H, CH_2_-indole), 2.07 (t, 2H, *J* = 7.4 Hz, CH_2_CON), 1.48 (m, 2H, CH_2_), 1.25 (m, 28H, 14 × CH_2_), 0.88 (t, 3H, *J* = 7 Hz, CH_3_); ^13^CNMR: δ (ppm) 175.1, 174.3 (2 × C=O , acid and amide), 136.1 (N-C of indole), 127.8 (C, indole), 123.2 (N-CH of indole), 122.2, 119.7 (2 × CH, Ph of indole), 118.4 (C of indole), 111.4, 109.5 (2 × CH, Ph of indole), 53.3 (CHN), 36.4(CH_2_-amide), 31.9 (CH_2_-indole), 29.7-27.1 (13 × CH_2_), 25.4 (CH_2_), 22.6 (CH_2_), 14.1 (CH_3_); ESI-MS: [M+1]^+^ m/z 471.2.

*N-Stearoyl-L-leucine* (**8**). Yield 49%; white solid; m.p. 95-97 °C; [α]${}_{D}^{20}$: −10.3° (*c* 1.0, CHCl_3_); ^1^H-NMR: δ (ppm) 5.9 (m, 1H, NH), 4.62 (m, 1H, CHN), 2.23 (t, 2H, *J* = 7.5 Hz, CH_2_CON), 1.72 (m, 2H, CH_2_), 1.62 (m, 2H, CH_2_), 1.60 (m, 1H, CH), 1.27 (m, 28H, 14 × CH_2_), 0.95 (d, 6H, *J* = 4.3 Hz, 2×CH_3_), 0.88 (t, 3H, *J* = 7 Hz, CH_3_); ^13^C-NMR: δ (ppm) 176.1, 174.1 (2 × C=O, acid and amide), 50.9 (CHN), 36.5 (CH_2_-amide), 31.9 (CH_2_), 29.7-29.2 (13 × CH_2_), 25.6 (CH_2_), 24.9 (CH_2_), 22.8 (CH), 22.6 (CH_3_), 21.9 (CH_3_), 14.1 (CH_3_); ESI-MS: [M-1]^+^ m/z 396.1.

*N-Stearoyl-L-cysteine* (**9**). Yield 30%; white solid; m.p. 146-148 °C; [α]${}_{D}^{20}$: +35.4° (*c* 1.0, CH_3_OH); ^1^HNMR (CD_3_OD): δ (ppm) 4.67 (m, 1H, CHN), 3.63 (m, 2H, CH_2_S), 2.25 (t, 2H, *J* = 7.2 Hz, CH_2_CON), 1.62 (m, 2H, CH_2_), 1.28 (m, 28H, 14 × CH_2_), 0.89 (t, 3H, *J* = 6.8 Hz, CH_3_); ^13^CNMR (CD_3_OD): δ (ppm) 176.5, 173.8 (2 × C=O, acid and amide), 50.1 (CHN), 37.3 (CH_2_-amide), 33.3 (CH_2_S), 31.1-30.6 (13 × CH_2_), 27.2 (CH_2_), 24.0 (CH_2_), 14.7 (CH_3_); ESI-MS: [M+1]^+^ m/z 388.0.

*N-Stearoyl-L-histidine* (**10**). Yield 56%; white solid; m.p. 127-129 °C; [α]${}_{D}^{22}$: +5.9° (*c* 1.0, CH_3_OH); ^1^H-NMR (CD_3_OD): δ (ppm) 8.46, 7.12 (m, 2H of imidazole), 4.5 (m, 1H, CHN), 3.04-3.22 (m, 2H, CH_2_-imidazole), 2.19 (t, 2H, *J* = 6 Hz, CH_2_CON), 1.51 (m, 2H, CH_2_), 1.28 (m, 28H, 14 × CH_2_), 0.89 (t, 3H, *J* = 6.9 Hz, CH_3_). ^13^C-NMR (CD_3_OD): δ (ppm) 176.1, 174.9 (2 × C=O, acid and amide), 135.1 (N-CH of imidazole), 133.1 (N-C of imidazole), 118.5 (NH-CH of imidazole), 54.6 (CHN), 37.4 (CH_2_-amide), 33.4 (CH_2_-imidazole), 31.1-29.7 (13 × CH_2_), 27.2 (CH_2_), 24.0 (CH_2_), 14.7 (CH_3_); ESI-MS: [M+1]^+^ m/z 422.1.

*N-Stearoyl-L-lysine* (**11**). Yield 50%; white solid; m.p. 85-87 °C; [α]${}_{D}^{20}$: −28.3° (*c* 1.0, CH_3_OH); ^1^H-NMR (CD_3_OD): δ (ppm) 4.19 (m, 1H, CHN), 3.17 (m, 2H, CH_2_N), 2.51 (m, 2H, CH_2_CON), 1.71 (m, 2H, CH_2_), 1.56 (2 × CH_2_), 1.28 (m, 30H, 15 × CH_2_), 0.89 (t, 3H, *J* = 6.8 Hz, CH_3_); ^13^C-NMR (CD_3_OD): δ (ppm) 170.3, 168.8 (2 × C=O, acid and amide), 48.3 (CHN), 31.6 (CH_2_NH_2_), 30.1 (CH_2_-amide), 29.2-28.7 (15 × CH_2_), 28.3 (CH_2_), 24.5 (CH_2_), 22.2 (CH_2_), 14.0 (CH_3_); ESI-MS: [M-1]^+^ m/z 411.2.

#### 3.2.2. Synthesis and spectroscopic data of N-stearoylamino acid methyl esters **12-14**

A mixture of the appropriate amino acid methyl ester (6.3 mmol) and octadecanoic acid 2,5-dioxo-pyrrolidin-1-yl ester (7 mmol) in dry pyridine (50 mL) was stirred at room temperature for 24 h. After evaporation of solvent, the residue was dissolved in dichloromethane (100 mL), and washed with HCl (1 M, 100 mL × 2), water (100 mL), 5% NaHCO_3_ (100 mL) and saturated NaCl (100 mL × 2). The organic layer was dried over anhydr. Na_2_SO_4_. After filtration and evaporation of the solvent *in vacuo*, the crude product was purified by recrystallization from methanol-chloroform.

*N-Stearoylglycine methyl ester* (**12**). Yield 24%; white solid; m.p. 82-84 °C; [α]${}_{D}^{20}$: 0° (*c* 1.0, CHCl_3_); ^1^H-NMR: δ (ppm) 5.97 (m, 1H, NH), 4.04 (d, 2H, *J* = 5.1 Hz, CH_2_N), 3.75 (s, 3H, CH_3_O), 2.23 (t, 2H, *J* = 7.5 Hz, CH_2_CON), 1.62 (m, 2H, CH_2_), 1.26 (m, 28H, 14 × CH_2_), 0.87 (t, 3H, *J* = 7 Hz, CH_3_); ^13^C-NMR: δ (ppm) 173.4, 170.7 (2 × C=O , acid methyl ester and amide), 52.4 (CH_3_O), 41.3 (CH_2_N), 36.5 (CH_2_-amide), 31.9 (CH_2_), 29.7-29.3 (12 × CH_2_), 25.6 (CH_2_), 22.8 (CH_2_), 14.1 (CH_3_); ESI-MS: [M+1]^+^ m/z 356.1

*N-Stearoyl-L-glutamic acid dimethyl ester* (**13**). Yield 23%; white solid; m.p. 85-87 °C; [α]${}_{D}^{19}$: +18° (*c* 1.2, CHCl_3_); ^1^H-NMR: δ (ppm) 6.15 (d, 1H, *J* = 7.4 Hz, NH), 4.64 (m, 1H, CHN), 3.77, 3.68 (s, 6H, 2 × CH_3_O), 2.39 (m, 2H, CH_2_), 2.21 (t, 2H, *J* = 7.4 Hz, CH_2_COO), 2.11 (m, 2H, CH_2_CON), 1.62 (t, 2H, CH_2_), 1.28 (m, 28H, 14 × CH_2_), 0.88 (t, 3H, *J* =6.8 Hz, CH_3_); ^13^C-NMR: δ (ppm) 173.3, 173.1, 172.5 (3 × C=O, acid methyl ester and amide), 52.5 (CHN), 51.8, 51.5 (2 × CH_3_O), 36.6 (CH_2_-amide), 31.9 (CH_2_-acid), 30.1 (CH_2_), 29.7-29.2 (12 × CH_2_), 27.4 (CH_2_), 25.6 (CH_2_), 22.7 (CH_2_), 14.1 (CH_3_); ESI-MS: [M+H]^+^ m/z 442.2.

*N-Stearoyl-L-phenylalanine methyl ester* (**14**). Yield 30%; grey solid; m.p. 70-72 °C; [α]${}_{D}^{22}$: −16.7° (*c* 1.0, CHCl_3_); ^1^H-NMR: δ (ppm) 7.28 (m, 3H of Ph), 7.12 (dd, *J_1_* = 1.5, *J_2_* = 6.7 Hz, 2H of Ph), 5.89 (d, 1H, *J* =7.6 Hz, NH), 4.92 (m, 1H, CHN), 3.75 (s, 3H, CH_3_O), 3.13 (m, 2H, CH_2_Ph), 2.18 (t, 2H, *J* = 6.8 Hz, CH_2_CON), 1.63 (m, 2H, CH_2_), 1.29 (m, 28H, 14 × CH_2_), 0.90 (t, 3H, *J* =7 Hz, CH_3_); ^13^C-NMR: δ (ppm) 173.7, 172.3 (2 × C=O , acid methyl ester and amide), 137.6 (*i*-C of Ph), 129.2 (2 × *o*-CH of Ph), 128.6 (2 × *m*-CH of Ph), 126.6 (*p*-CH of Ph), 64.3 (CHN), 52.8 (CH_3_O), 37.0 (CH_2_Ph), 36.8 (CH_2_-amide), 31.9 (CH_2_), 29.7-29.1 (12× CH_2_), 25.7 (CH_2_), 22.7 (CH_2_), 14.2 (CH_3_); ESI-MS: [M+1]^+^ m/z 446.3.

#### 3.2.3. Synthesis and spectroscopic data of *N*-palmitoyl-l-tyrosine (**15**)

Hexadecanoic acid 2,5-dioxo-pyrrolidin-1-yl ester was synthesized following the synthetic route of octadecanoic acid 2,5-dioxo-pyrrolidin-1-yl ester from palmitic acid instead of stearic acid. Then **15** was prepared from hexadecanoic acid 2,5-dioxo-pyrrolidin-1-yl ester instead of octadecanoic acid 2,5-dioxo-pyrrolidin-1-yl ester by the same procedure of **1-11**. Yield 45%; white solid; recrystallization from n-hexane; m.p. 115-117 °C; [α]${}_{D}^{22}$: +24.2° (*c* 1.0, CH_3_OH); ^1^H-NMR (CD_3_OD): δ (ppm) 7.03 (m, 2H of Ph), 6.73 (m, 2H of Ph), 4.62 (m, 1H, CHN), 2.8-3.09 (m, 2H, CH_2_Ph), 2.15 (t, 2H, *J* = 7.5 Hz, CH_2_CON), 1.48 (m, 2H, CH_2_), 1.28 (m, 24H, 12× CH_2_), 0.89 (t, 3H, *J* = 7 Hz, CH_3_); ^13^C-NMR (CD_3_OD): δ (ppm) 176.4, 175.3 (2 × C=O , acid and amide), 157.6 (C-OH of Ph), 131.5 (*i*-C of Ph), 129.4, 122.8 (2 × *o*-CH of Ph), 116.6 (2 × *m*-CH of Ph), 55.4 (CHN), 37.9 (CH_2_Ph), 37.1 (CH_2_-amide), 33.4 (CH_2_), 31.1-30.4 (10 × CH_2_), 27.2 (CH_2_), 24.0 (CH_2_), 14.7 (CH_3_); ESI-MS: [M+1]^+^ m/z 420.1.

#### 3.2.4. Synthesis and spectroscopic data of N-lauroyl-l-tyrosine (**16**)

Dodecanoic acid 2,5-dioxo-pyrrolidin-1-yl ester was synthesized following the synthetic route of octadecanoic acid 2,5-dioxo-pyrrolidin-1-yl ester from lauric acid instead of stearic acid. Then **16** was prepared from dodecanoic acid 2,5-dioxo-pyrrolidin-1-yl ester instead of octadecanoic acid 2,5-dioxo-pyrrolidin-1-yl ester by the same procedure of **1-11**. Yield 50%; white solid; recrystallization from *n*-hexane; m.p. 95-97 °C; [α]${}_{D}^{22}$: +17.4° (*c* 1.0, CH_3_OH); ^1^H-NMR (CD_3_OD): δ (ppm) 6.99 (m, 2H of Ph), 6.73 (m, 2H of Ph), 4.62 (m, 1H, CHN), 2.8-3.09 (m, 2H, CH_2_Ph), 2.14 (t, 2H, *J* = 7.5 Hz, CH_2_CON), 1.48 (m, 2H, CH_2_), 1.27 (m, 16H, 8 × CH_2_), 0.88 (t, 3H, *J* = 6.8 Hz, CH_3_); ^13^C-NMR (CD_3_OD): δ (ppm) 176.6, 175.4 (2C, acid and amide C=O), 157.8 (C-OH of Ph), 131.7 (*i*-C of Ph), 129.6, 123.1 (2 × *o*-CH of Ph), 116.6 (2 × *m*-CH of Ph), 55.6 (CHN), 38.2 (CH_2_Ph), 37.3 (CH_2_-amide), 33.6 (CH_2_), 31.3-30.6 (6 × CH_2_), 27.3 (CH_2_), 24.2 (CH_2_), 14.9 (CH_3_); ESI-MS: [M+H]^+^ m/z 364.0.

#### 3.2.5. Synthesis and spectroscopic data of *N*-stearoyl-l-tyrosinol (**17**)

To *L*-tyyrosine (1.81 g, 10 mmol) and NaBH_4_ (0.91 g, 24 mmol) dissolved in THF (50 ml) were added dropwise I_2_ (2.54 g, 10 mmol) in THF (7.5 mL) during 10 min. Then, the mixture was refluxed for 5 h. It was then cooled to room temperature, and MeOH (5 mL) was added. The solvent was evaporated and the residue dissolved in 2 M HCl (10 mL). After removal of the solvent *in vacuo*, the residue was then suspended in EtOH (30 mL) at 40 °C and the suspension filtered. The filtrate was concentrated until crystals appear and was kept at 5 °C overnight. The resulting crystals were isolated by filtration, washed with EtOH, and dried *in vacuo* to yield 45% of L-tyrosinol hydriodide as colorless crystals: m.p. 214-216 °C. Then **17** was prepared from L-tyrosinol hydriodide instead of L-tyrosine by the same procedure of **1-11.** Yield 30%; white solid; recrystallization from n-hexane; m.p. 121-123 °C; [α]${}_{D}^{22}$: +27.1° (*c* 1.0, CH_3_OH); ^1^H-NMR: δ (ppm) 7.28 (m, 2H of Ph), 7.22 (m, 2H of Ph), 5.78 (d, 1H, *J* = 7.6 Hz, NH), 4.17 (m, 1H, CHN), 3.66-3.58 (m, 2H, CH_2_O), 2.86 (m, 2H, CH_2_Ph), 2.12 (t, 2H, *J* = 7.5 Hz, CH_2_CON), 1.53 (m, 2H, CH_2_), 1.25 (m, 28H, 14 × CH_2_), 0.88 (t, 3H, *J* = 7.1 Hz, CH_3_); ^13^C-NMR: δ (ppm) 173.9 (CONH), 155.6 (C-OH of Ph), 137.7 (*i*-C of Ph), 129.1, 128.6 (2 × *o*-CH of Ph), 126.6 (2 × *m*-CH of Ph), 63.3 (CH_2_OH), 52.8 (CHN), 37.0 (CH_2_Ph), 36.8 (CH_2_-amide), 31.9 (CH_2_), 29.7-29.1 (12× CH_2_), 25.7 (CH_2_), 22.7 (CH_2_), 14.1 (CH_3_); ESI-MS: [M+H]^+^ m/z 434.3.

### 3.3. Neuroprotective effects of the target compounds on rat brain slices in vitro injury models

The slices were prepared as described by Bickler with modification [26]. Whole brain from male Sprague-Dawley rats (about 100 g ± 50 g) were obtained after decapitation and quickly removed to ice-cold oxygenated (95 % O_2_/5 % CO_2_) normal artificial cerebrospinal fluid (ACSF), which had the following composition (in mM): NaCl 119, KCl 2.5, CaCl_2_ 2, MgSO_4_ 1, NaH_2_PO_4_ 1.25, NaHCO_3_ 26.2, glucose10 (final pH 7.4). Brain slices (400 μm thick) were prepared using a vibrating tissue slicer (ZQP-86, Xiangshan, Zhejiang, China), then transferred to a ‘slice saver’ containing continuously-oxygenated normal ACSF at 32-34 °C for 90 min recover. After that, brain slices were transferred to experimental chambers and randomly assigned to one of following groups: control group, in which slices were immersed in oxygenated ACSF at 34 °C; OGD group, in which slices were made anoxic by switching the ACSF into the glucose-free ACSF equilibrated with 95% N_2_/5% CO_2_. After 10 min, slices were reoxygenated in ACSF for 2 h; OGD + compound group, in which slices were incubated with different 10 μM target compounds (or 1~20 μΜ compound **5**) for 30 min prior to and during OGD application; glutamate group, in which slices were subjected to 1 mM glutamate in magnesium-free artificial cerebrospinal fluid for 15 min (which had the following composition [in mM]: NaCl 143, KCl 5.4, CaCl_2_ 1.8, NaH_2_PO_4_ 1.0, HEPES 2.4, glucose 5.6 pH = 7.4). Slices were reoxygenated in ACSF for 2 h; Glu + **5** group, in which slices were incubated with 1–20 μΜ **5** for 30 min prior to and during Glu application; H_2_O_2_ group, in which slices were subjected to 2 mM H_2_O_2_ for 30 min; H_2_O_2_ + **5** group, in which slices were incubated with different concentrations of **5** (1–20 μM) 30 min prior to and during H_2_O_2_ application.

After insult, slices were immersed into 2% TTC solution at 37 °C for 30 min and the wet weight was measured after rinsing twice with saline. A mixture of ethanol and dimethylsulfoxide (50:50) was added at a rate of 20 mL per 1 g of slices. After 24 h extraction in dark, the extracted liquid was added to 96-well plates (200 μL per well), the absorbance (*A*) at 490 nm of each well was measured by the ELISA reader (Elx800^uv^, Bio-TEK). The viability of brain slices was evaluated from the following equation: % tissue activities = 100% × (*A*_injury_ / *A*_control_).

## 4. Conclusions

In summary, we have synthesized a series of *N*-stearoylamino acids and their analogues by a facile route, and evaluated their activity against cerebral ischemia *in vitro*. Among these compounds, **4**, **5** and **6** elicited higher activity in protecting rat brain slices against OGD insult. According to the preliminary SAR study, the neuroprotective activity of these lipoamino acids might be related with the fatty-acyl moieties of the tested compounds and the presence of a free carboxylic and hydroxyl groups on the branched chain of the amino acids. The evaluation of neuroprotective effects of **5** with three models implies that certain **LA**s could protect brain slices against OGD as well as H_2_O_2_ insult. Therefore, they might serve as candidate for the amelioration of cerebral ischemia and other cerebral insults.

## Figures and Tables

**Table 1:** Library of **LA**s.

Chemical Name	R
**1**. *N*-stearoyl-L-phenylalanine	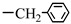
**2**. *N*-stearoyl-DL-phenylalanine	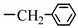
**3**. *N*-stearoyl-L-proline	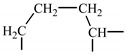
**4**. *N*-stearoyl-L-tyrosine	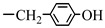
**5**. *N*-stearoyl-L-serine	—CH_2_OH
**6**. *N*-stearoyl-L-threonine	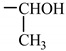
**7**. *N*-stearoyl-L-tryptophan	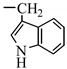
**8**. *N*-stearoyl-L-leucine	—CH(CH_3_)_2_
**9**. *N*-stearoyl-L-cysteine	—CH_2_SH
**10**. *N*-stearoyl-L-histidine	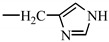
**11**. *N*-stearoyl-L-lysine	—(CH_2_)_4_NH_2_
**12**. *N*-stearoylglycine methyl ester	—H
**13**. *N*-stearoyl-L-glutamic acid dimethyl ester	—(CH_2_)_2_COOCH_3_
**14**. *N*-stearoyl-L-phenylalanine methyl ester	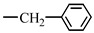
**15**. *N*--palmitoyl-L-tyrosine	—COOH, n = 14
**16**. *N*- lauroyl-L-tyrosine	—COOH, n = 10
**17**. *N*-stearoyl-L-tyrosinol	—CH_2_OH, n = 16
